# *In vitro* and *in vivo* antitumor effects of lupeol-loaded galactosylated liposomes

**DOI:** 10.1080/10717544.2021.1905749

**Published:** 2021-04-07

**Authors:** Jun Zhang, Xixi Hu, Guohua Zheng, Hui Yao, Huali Liang

**Affiliations:** a Department of Pharmacy, Xiangyang No. 1 People’s Hospital, Hubei University of Medicine, Xiangyang, Hubei Province, China; b College of Pharmacy, Hubei University of Chinese Medicine, Wuhan, Hubei Province, China; c College of Pharmacy, Hubei University of Science and Technology, Xianning, Hubei Province, China; d Nursing Department, Xiangyang Central Hospital, Hubei University of Arts and Science, Xiangyang, Hubei Province, China

**Keywords:** Gal-lupeol liposomes, encapsulation efficiency, Nile Red, high-pressure tail vein, liver targeted

## Abstract

Lupeol liposomes, modified with Gal-PEG-DSPE, were developed following a thin-film dispersion method. Then, the morphology, physicochemical properties, and *in vitro* release properties of those liposomes were investigated. The scanning electron microscopic images showed that most of the liposomes were spherical particles; they were similar in size and uniformly dispersed. Both lupeol liposomes and Gal-lupeol liposomes exhibited an average particle size of about 100 nm. The encapsulation efficiency was greater than 85%. The encapsulation efficiency of lupeol liposome and Gal-lupeol liposome, stored with 15% sucrose as glycoprotein for 6 months, was higher than 80%; although the particle size increased, they remained within 200 nm. The cell-uptake study demonstrated that the Gal-lupeol-liposome uptake efficiency was the highest in HepG2 cells. The HepG2 cells treated with the Gal-lupeol liposomes had higher apoptotic efficiency than the lupeol liposome and free lupeol. After HepG2 cells were treated with Gal-lupeol liposome, the expressions of AKT/mTOR-related proteins (p-AKT308 and p-AKT473) were also significantly reduced than the lupeol-liposome and free lupeol group. The *in vivo* targeting studies showed that Gal-NR-L exhibited liver-targeting effects on FVB mice. The pharmacodynamic study was performed by transfecting AKT and c-MET via the high-pressure tail vein of FVB mice. After Gal-lupeol-L administration, the liver index and liver weight of mice were less than those non-targeted group. The histopathological study showed that the lobular structure in the mice liver was clearer, the vacuoles were more obvious, and the cytoplasm was more abundant after Gal-lupeol-L administration. Also, the qRT-PCR study showed that AFP, GPC3, and EpCAM mRNA expression levels were significantly lower than those non-targeted lupeol-liposomes.

## Introduction

Hepatocellular carcinoma (HCC) is the sixth most common cancer worldwide. HCC is routinely treated via surgical resection, liver transplantation, and percutaneous ablation (Varela et al., 2003; Abou-Alfa & Venook, 2008; Thomas et al., 2008). Unfortunately, most of these treatments exhibited many side effects, and their application is limited by resistance to treatment and disease recurrence (Llovet et al., 2003). Therefore, a new drug or a novel drug delivery system should be developed to solve these problems.

In the past few decades, numerous studies have confirmed that lupeol plays a significant anti-tumor role in various tumor cell lines and cancer models (Chaturvedi et al., 2008; Saleem, 2009; Siddique & Saleem, 2011). Also, lupeol can inhibit the self-renewal ability of liver tumor-initiating cells (Lee et al., 2011) and induce apoptosis of human epidermoid cancer cells by negatively regulating the mitochondrial AKT/PKB and NF-kB signaling pathways (Prasad et al., 2009). Therefore, a new drug carrier should be developed to improve its properties and targeting effect.

PEGylated liposome (PLS) is a potential carrier for anticancer chemotherapeutic agents (Zhou et al., 2012; Yu et al., 2013; Allen & Cullis, 2013; Bozzuto & Molinari, 2015) because liposomes can enhance the tissue permeability and retention effect (EPR effect) (Allen & Cullis, 2013; Bozzuto & Molinari, 2015). Additionally, liposome ligand modification can enhance the drug accumulation at the cancer cells, thus significantly improving its efficacy.

Sialic acid glycoprotein receptor (ASGPR) is a promising drug target for the HCC treatment because of its high expression on the surface of HCC cell line (Poelstra et al., 2012; Nair et al., 2019; Zhang et al., 2019). Also, lactoferrin (LF) and ASGPR can bind in a non-dependent way with high affinity. It is a type of mammalian cationic iron-binding glycoprotein, belonging to the TF family (Mcabee et al., 1998; Mcabee et al., 2000; Ward & Conneely, 2004; Mishra et al., 2013). Through its specific binding, LF has been successfully applied to gene transfer (Abe et al., 2007; Weeke-Klimp et al., 2007; Pathak et al., 2008; Wei et al., 2012). In a previous study, we have successfully constructed the PLS system. The results show that PLS can significantly target HCC, and its toxicity is low. However, it is difficult to determine whether the use of lupeol liposomes containing targeting carriers can enhance the accumulation of drugs in liver cancer cells and enhance the antitumor effect. Therefore, in this work, we will verify the targeting efficiency of Gal-lupeol-L and the antitumor effect *in vivo* and *in vitro*.

This study has been aimed at establishing the Gal-lupeol-L system and studying its targeting and antitumor effects on HCC. Gal-lupeol-L was prepared via a thin-film method and characterized in detail. To establish the targeting effect, this system was evaluated *in vitro* by a live cell imager. To examine the antitumor effect of Gal-lupeol-L, we administered Gal-lupeol-L on HepG2 hepatoma cells to study the targeting efficiency of liposomes and the proteins of the related pathways. To further verify the antitumor ability, targeting efficiency, and molecular mechanism of lupeol liver targeting liposome in the optimal dosage of AKT/c-Met-induced HCC, a mouse HCC model was constructed using gene transfection technology via high-pressure injection.

## Materials and methods

Lupeol was obtained from Chengdu Pusi Biotechnology Co., Ltd. HSPC was procured from Shanghai AVT Co., Ltd. Cholesterol, Nile Red were received from Solarbio Biotechnology Co., Ltd. Galactose-PEG-DSPE was purchased from Xi'an Ruixi Biotechnology Co., Ltd. Fluorescein isothiocyanate (FITC) was provided by Sigma-Aldrich (St. Louis, MO). The other reagents and solvents used were of analytical grade. Professor Xin Chen's laboratory (University of California, San Francisco, CA) provided pT3-EF1α-HA-myr-AKT, pT3-EF1α-V5-c-Met, and pCMV/sleeping beauty transposable (SB). The FVB/N mice were supplied by Beijing Vital River Laboratory Animal Technology Co., Ltd.

### Preparation of lupeol-loaded galactose-PEG-DSPE liposomes

Lupeol-loaded galactose-PEG-DSPE liposomes (Gal-L) were prepared following the thin-film dispersion method. Briefly, lupeol, HSPC, cholesterol, and galactose-PEG-DSPE were dissolved at a molar ratio of 1:10:2:2 in the methanol/chloroform (3:1) solvent. The organic solvents were evaporated to dryness using a rotary vacuum evaporator, and the film was hydrated at 60 °C for 20 min with phosphate-buffered saline (PBS) (pH 7.4). The suspension of lipids was ultrasonicated by an ultrasound cell crusher for 10 min. Finally, the liposome suspension was filtered three times through a 0.22 µm microporous filter to remove the free lupeol. Lupeol liposomes (lupeol-L) were prepared similarly, except that galactose-PEG-DSPE was removed.

#### Characterization of liposome

The particle size and polydispersity index of Gal-L and lupeol-L were measured using a Malvern particle size analyzer (Nano ZS-90; Malvern, UK). The morphology of liposomes was observed under a scanning electron microscope (SEM, JSM-6510LV, JEOL, Japan). Briefly, 10 μL liposome solution was placed on a coverslip and quickly dried with nitrogen, then examined in a SEM system.

### Entrapment efficiency

Lupeol were determined using an HPLC method. Using the LC1260 HPLC system (Agilent, USA), on an Zorbax C_18_ column (1.8 μm,150 × 2.1 mm), column temperature set to 30 °C. The detection wavelength is 210 nm, the mobile phase was methanol: water (94:6), and the flow rate was 1 mL min^−1^. Samples were diluted in methanol and taken 20 μL of the solution for the test. The encapsulation efficiency of the liposomes was evaluated following the membrane method (free lupeol could not pass the 0.22 µm microporous filter membrane, but liposome could pass because of its solubility). The absorption was measured at 210 nm after the liposomes were cleaved in methanol. The encapsulation efficiency of liposomes was calculated using the following formula

EE% = (amount of encapsulated lupeol in liposome)/(amount of lupeol added totally)

### Freeze drying and stability

The liposomes were prepared following the thin-film dispersion method based on the optimal mixing process. Sucrose, trehalose, mannitol, and lactose were used as lyophilization protective agents. We investigated the lyophilization protection effect of Gal-lupeol-L and lupeol-L at different dosages. Briefly, we dissolved various lyophilization protective agents at 7, 10, and 15% of the total lipid mass in the prepared drug-loaded liposomes. After complete dissolution, we prefreezed them for 24 h at −20 °C. After freeze-drying, we obtained a lyophilized powder of liposomes, and the encapsulation efficiency was calculated after reconstitution. Lyophilized liposome samples were placed in the refrigerator for 6 months, the appearance of the liposomes was observed, and the encapsulation efficiency and particle size of the samples after reconstitution were measured.

#### Cell-uptake study

For visualizing the endocytosis of the formulation in the HepG2 cells, 5% (w/w) fluorescein isothiocyanate (FITC), a fluorescence marker with green fluorescence, was encapsulated in liposomes as a test drug. The samples were prepared following a similar process to that of the liposomes describe above. The HepG2 cells were seeded in six-well plates at a density of 1.0 × 10^6^ cells/well and cultured at 37 °C under 5% CO_2_ for 24 h. The medium was replaced by test sample solutions: FITC, FITC-loaded lupeol liposome, and FITC-loaded DSPE-PEG-lupeol liposome at a FITC dose of 5 μM. The cells were incubated for another 2 h and then washed three times with ice-cold PBS to terminate the uptake. Finally, the cells were photographed by EVOS FL, an auto cell imaging system (Thermo Fisher).

#### Apoptosis

The HepG2 cells were seeded in a six-well cell culture plate at a density of 1 × 10^6^ cells/well and incubated overnight with complete medium containing calf serum. When 80% of the cells had adhered to the plate, the original medium was removed, and 2 mL of each 30 µmol free lupeol, lupeol-L, and Gal-lupeol-L were added. Additionally, a blank control group, a single-yang group, and a normal cell group were set and collected after 24 h. The medium was digested with trypsin without EDTA, and the cells were harvested after centrifuging for 5 min. The digestion was terminated with the collected medium. The medium was centrifuged to remove the medium. The cells were washed twice with pre-chilled PBS and centrifuged at 2000 rpm for 5 min to collect the cells. Then, PBS was aspirated, 100 μL of 1 × binding buffer was added to resuspend the cells, 5 μL of FITC and 10 μL of PI staining solution were added and mixed gently; the mixture was protected from light to react at room temperature for 10–15 min; 400 μL of 1 × binding buffer was added and mixed well. After placing on ice, the samples were detected by flow cytometry within 1 h.

### *In vitro* pharmacodynamic studies

The HepG2 cells were seeded in a 10-cm culture plate at a density of 7 × 10^6^ cells/well and cultured overnight with complete medium containing calf serum. When 70% of the cells had adhered to the plate, plasmid pT3-EF1α-HA-myr-AKT and pT3-EF1α-V5-c-Met were transferred to the HepG2 cells. After 24 h of culture, the original medium was removed and washed three times with PBS. Then, 10 mL of calf serum-free medium containing different preparations (lupeol, lupeol-L, and Gal-lupeol-L) was added at a concentration of 60 µmol/L. Finally, Western blotting was performed. The expression was visualized by a G-Box gel imaging system (Syngene).

### *In vivo* imaging

The FVB/N mice were modeled by high-pressure tail vein transfection technology, and a saline solution (AKT and c-MET: Sleeping Beauty (SB) was 25:1) was injected at a dose of 10% body weight through the tail vein within 5–9 s. Nile red (NR) replaced lupeol to prepare liposomes. After 4 weeks, free NR, NR-L, and Gal-NR-L were injected into the FVB mice through the tail vein. Under the fluorescence intensity of EM748/780 nm, the anesthetized animals were monitored for the real-time changes by a small animal *in vivo* imaging system at 1, 3, 5, and 8 h. Then, the FVB tumor-bearing mice were authorized, and their organs (heart, liver, spleen, lung, and kidney) were dissected and scanned with a small animal imaging system to observe the organ enrichment at each time point.

### *In vivo* pharmacodynamic study

Wild-type FVB/N mice (age: about 7 weeks) were selected. The mice were weighed, and then, they were fixed with a mouse holder until the veins of the mice became prominent. A saline solution (AKT and c-MET: Sleeping Beauty (SB) was 25:1) was injected at a dose of 10% body weight through the tail vein within 5–9 s. Subsequently, the transfected mice were randomly divided into three groups (*n* = 9), and the standard diet was fed for three weeks. No transfected mice were used as the blank control group (*n* = 9), and the same standard diet was fed. From the fourth week after modeling, approximately 0.2 mL of each of PBS, free lupeol, lupeol-L, or Gal-lupeol-L solution were injected through the tail vein, and a dose of 10 mg/kg body weight was injected every other day for three weeks. At the end of the treatment, the mice were euthanized, the liver tissues were removed, and the morphology of the liver tissues of different groups was observed, photographed, then washed, dried, and weighed. A part of the stripped liver tissue was fixed in 4% paraformaldehyde for hematoxylin and eosin (H & E) staining, and the remaining liver tissue was frozen in a −80 °C refrigerator for tumor marker determination. Using RT-PCR analysis, we calculated the mRNA expression levels of AFP, GPC3, and Epcam in the livers of different groups of mice.

#### Statistical analysis

All experimental data in this study have been expressed as the mean ± SD. The statistical analysis was performed using SPSS22.0. The comparison between the two groups was performed by *t*-test, and the comparison between multiple groups was performed by ANOVA (analysis of variance). The difference between the means was considered significant at *p* < 0.05.

## Results

### Preparation and characterization of liposomes

**Figure 1.**
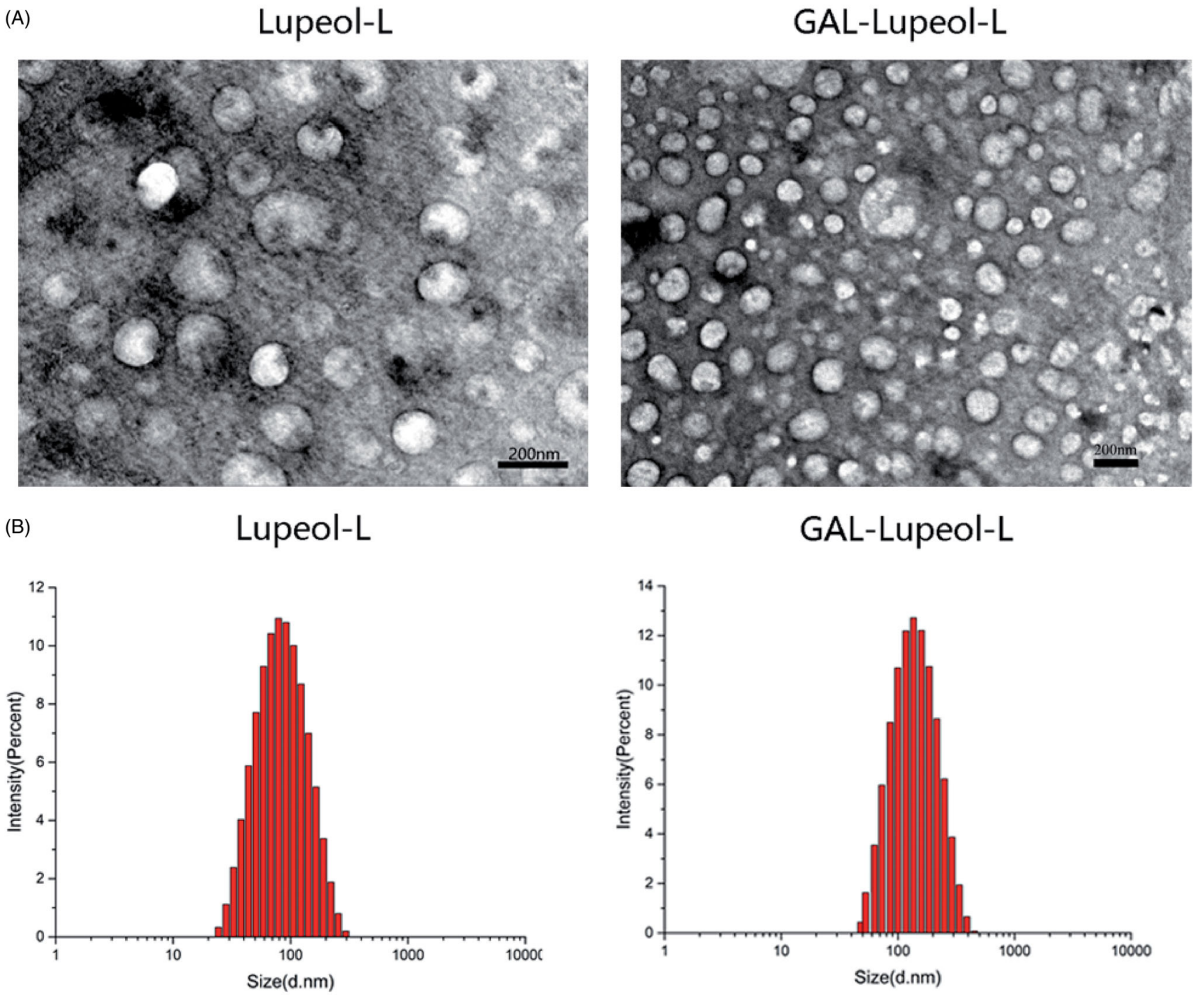
(A) SEM image of liposome; (B) dynamic light scattering of liposomes.

To achieve the liver-targeted delivery of lupeol, we prepared a galactose-modified long-circulating liposome (Gal-lupeol-L). Since galactose-PEG-DSPE in liposomes affects the size of the liposomes and the uptake of lupeol cells key factors, we screened for the best process. When the phosphoro-biliary ratio was 7:1, the drug-lipid ratio was 1:10, prescription quality ratio of Gal-PEG-DSPE was 10%, the encapsulation efficiency and cell uptake of liposomes were highest. As a control, lupeol-L was prepared at the above ratio, and its particle size was 97.23 ± 0.6 nm, which was significantly smaller than that of Gal-lupeol-L because galactose-PEG-DSPE significantly increased the particle size of Gal-lupeol-L. Figure 1(A) shows the smooth spherical morphology of Gal-lupeol-L, which was observed under an TEM, and proved that Gal-lupeol-L liposomes with uniform size were successfully assembled. Figure 1(B) shows the particle size distribution of liposomes, measured by dynamic light scattering, showing a normal distribution. Lupeol was coated with galactose-modified PLSs, and its water solubility was significantly enhanced.

### Investigation of the lyophilization protective agent and its stability

**Figure 2.**
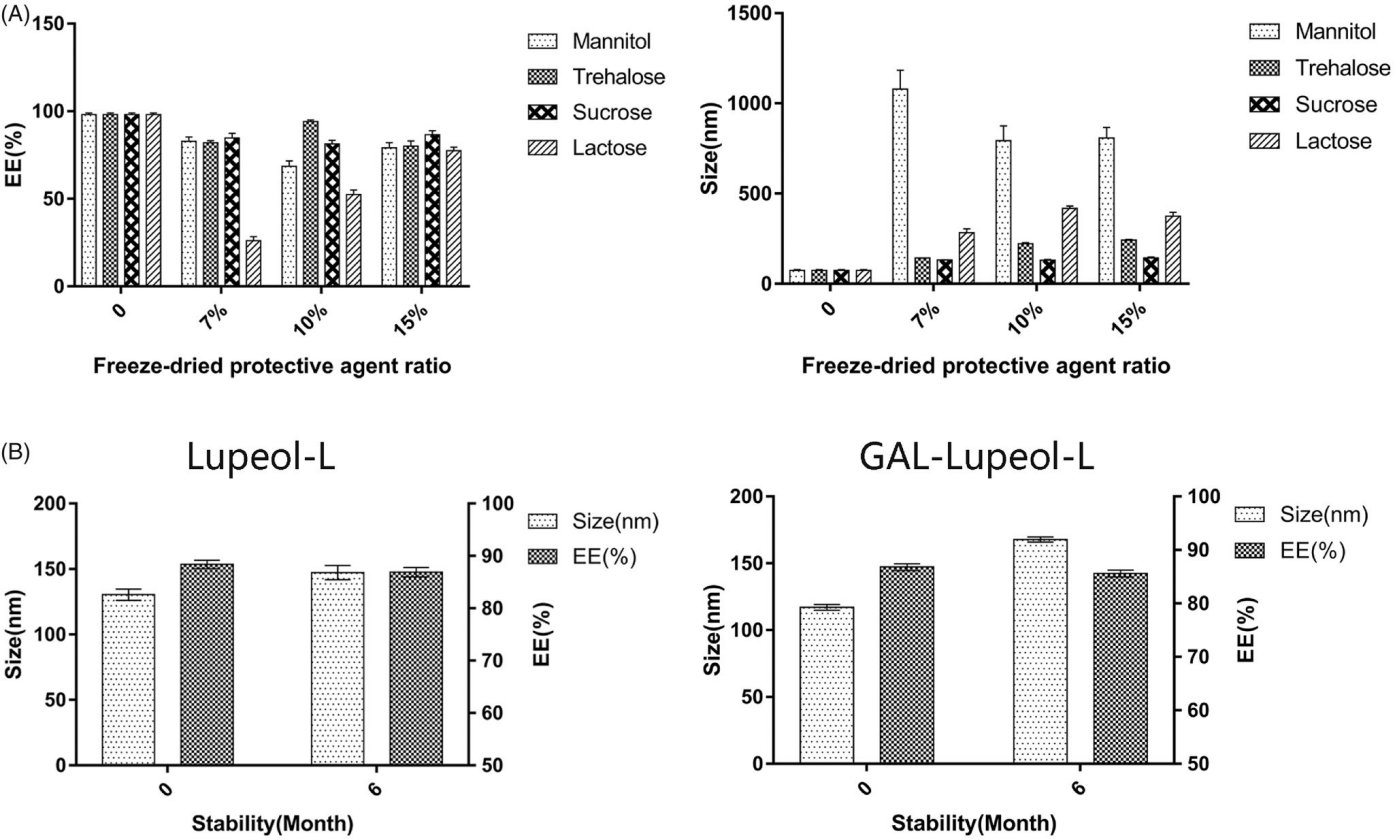
Freezing and stability of liposomes.

Figure 2 shows that the particle size of the liposomes, after reconstitution of mannose and lactose, is much larger than 200 nm, and only 7% of trehalose has a proportional particle size below 200 nm. The encapsulation efficiency is the highest at 15%. So, 15% sucrose solution has been selected as the lyophilization protective agent. Lupeol-L and Gal-lupeol-L were freeze-dried and stored in the refrigerator. The stability results showed that the appearance of the preparation, stored at 4 °C for 6 months, did not change significantly, and the particle size also increased with time. The leakage rate is less than 2%, indicating that the lyophilized preparation of lupeol liposomes is relatively stable at 4 °C for 6 months.

### *In vitro* cellular uptake study

**Figure 3.**
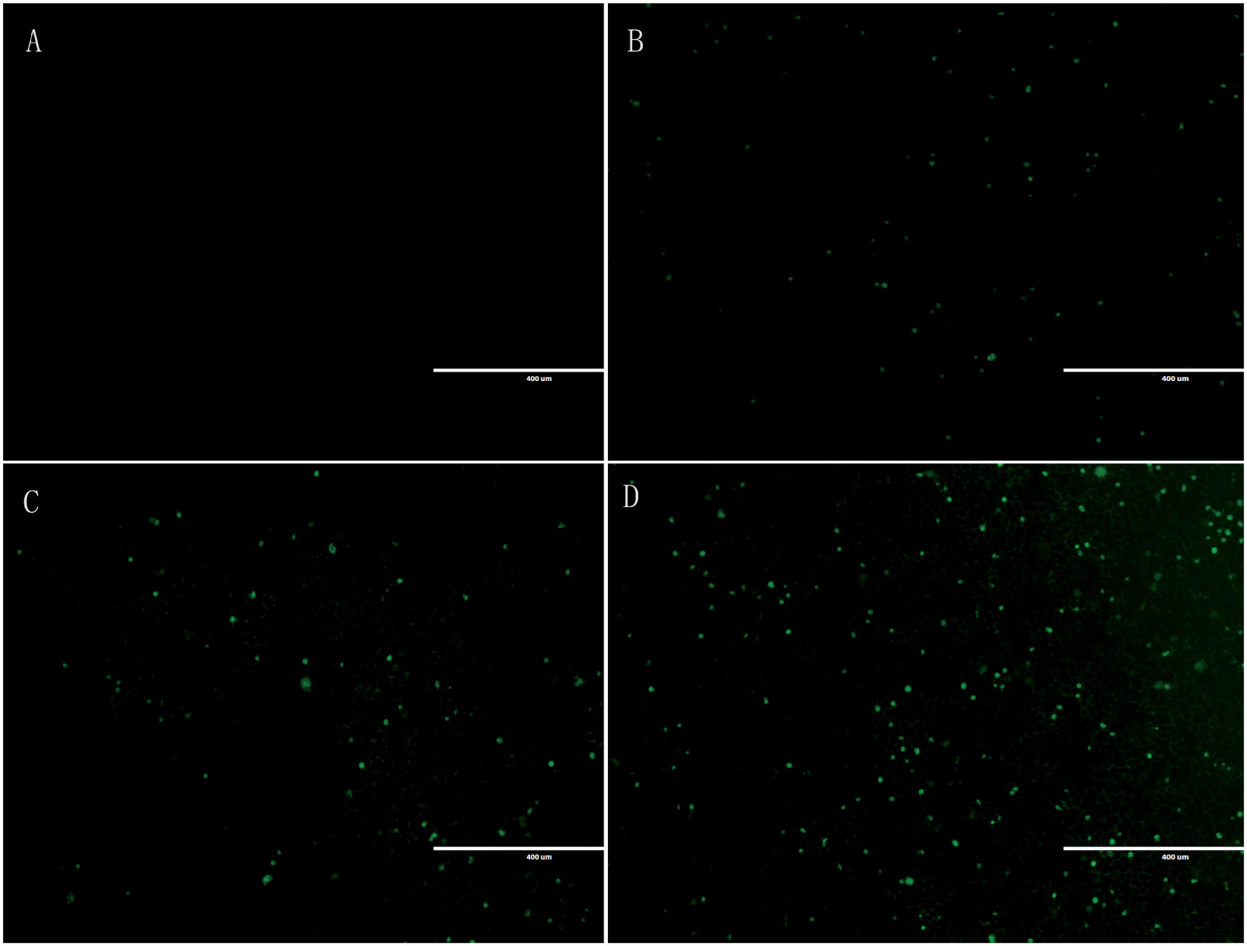
*In vitro* cellular uptake study. (A) Control; (B) free lupeol; (C) lupeol liposomes; (D) Gal-lupeol liposomes.

The cellular uptake of liposomes by HepG2 cells was analyzed by EVOS FL, an auto cell imaging system (Thermo Fisher, USA). FITC (the fluorescent indicator) was entrapped into the liposome aqueous phase. Figure 3 shows that compared to the blank group, the dosage intensity of each dosage form group is significantly higher than that of the blank group; the intake intensity of the Gal-FITC-L group is higher than that of the free drug group and the nontargeted drug group, suggesting that Gal-FITC-L has a certain targeting effect compared to the blank group and other dosage groups.

#### Apoptosis

**Figure 4.**
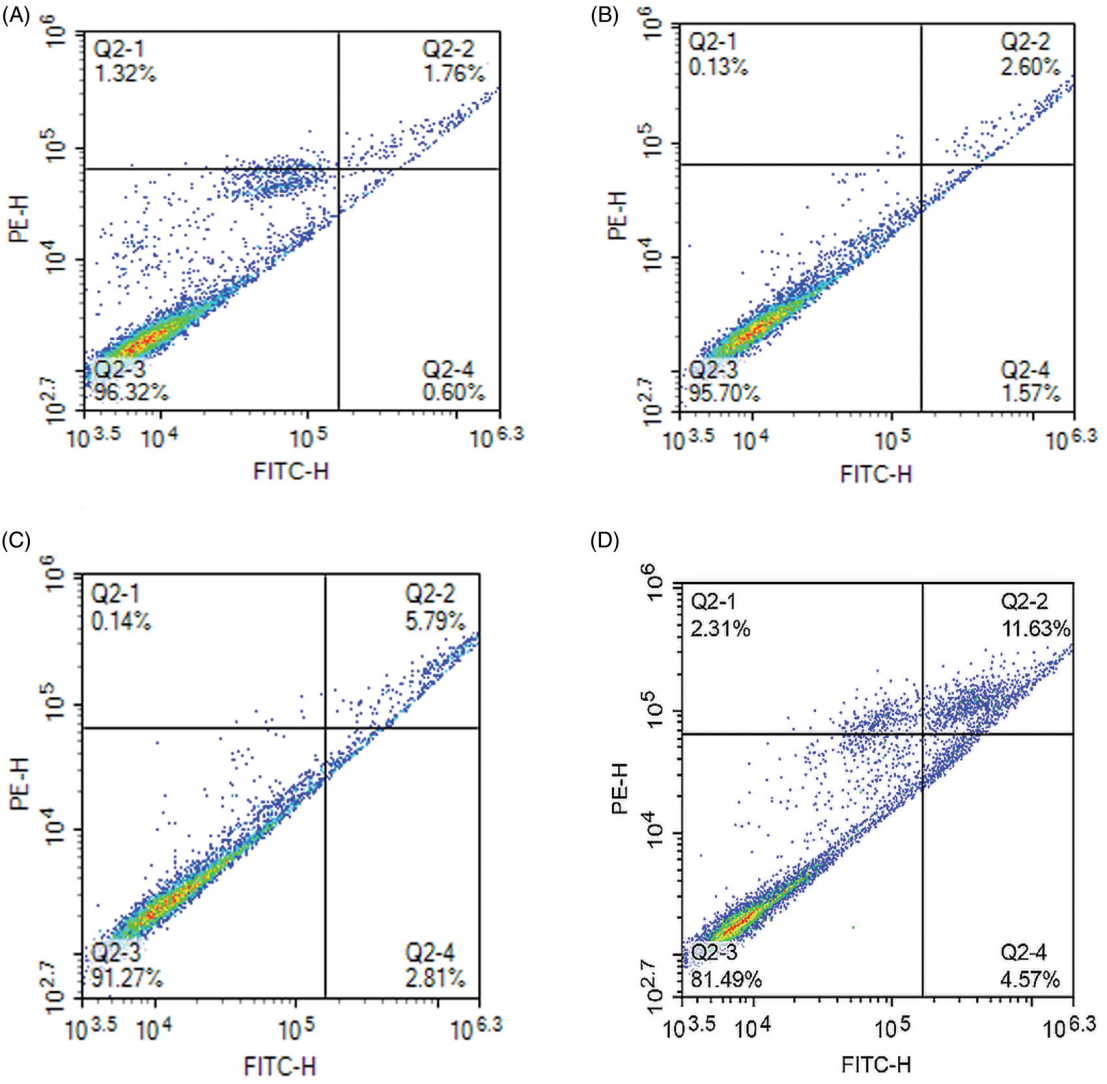
Effects of apoptosis on HepG2 cells. (A) Control; (B) free lupeol; (C) lupeol liposomes; (D) Gal-lupeol liposomes.

Figure 4 shows that the number of living cells in the negative blank control group is 96.32%, while the number of living cells in the free lupeol, lupeol-L, Gal-lupeol-L groups are 95.7, 91.27, 81.49%, and the Gal-lupeol-L group shows the lowest number of viable cells. Comparing the number of pro-apoptotic cells in various groups of lupeol, we found that the effect of promoting late apoptosis was stronger than that of promoting early apoptosis. Free lupeol, lupeol-L, Gal-lupeol-L group promoted early apoptosis. The numbers of cells were 1.57, 2.81, and 4.57%, respectively, and the numbers of cells, promoting advanced apoptosis, were 2.6, 5.79, and 11.63%, respectively, indicating that Gal-lupeol-L promoted apoptosis more efficiently than free lupeol and lupeol-L.

### Molecular mechanism of apoptosis

**Figure 5.**
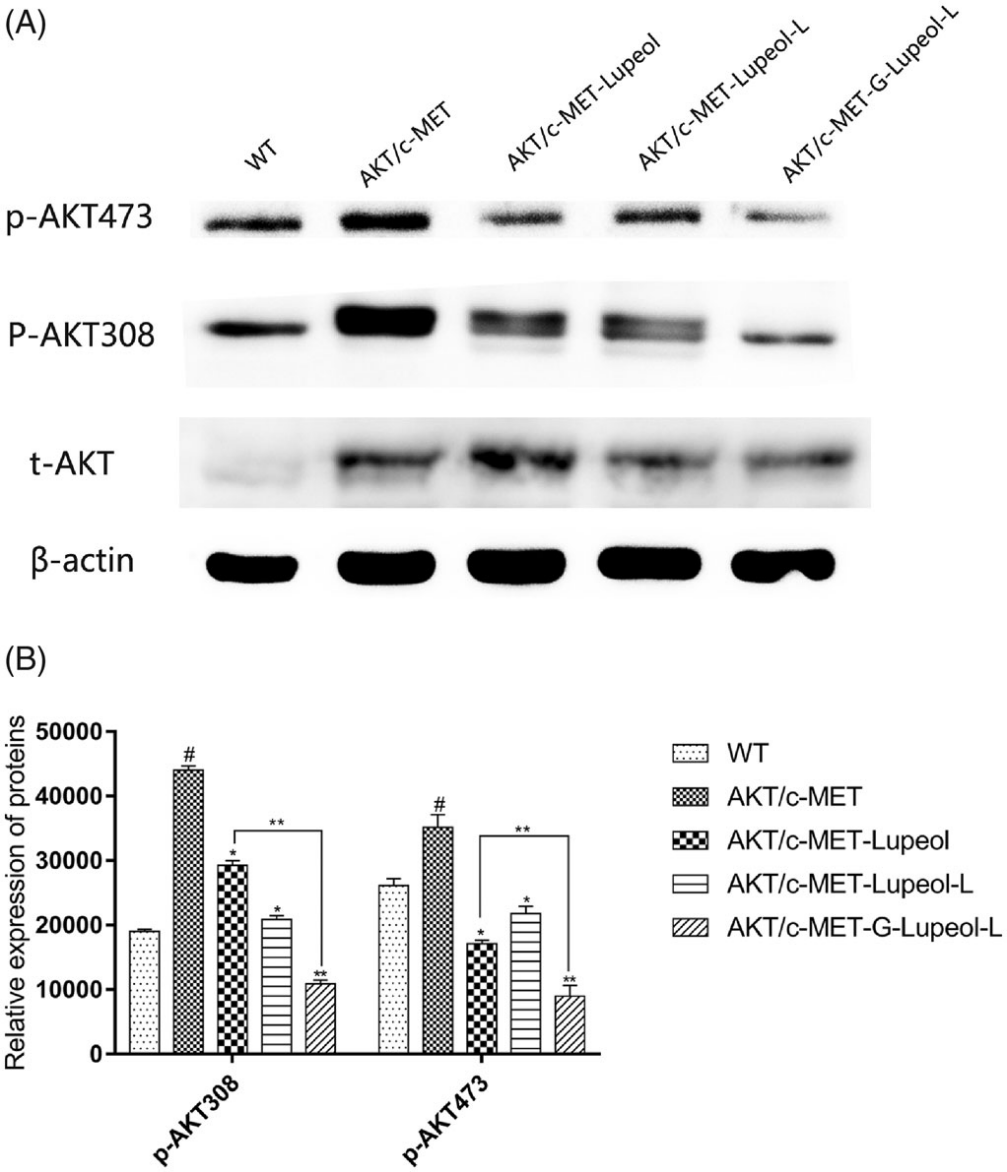
Molecular mechanism of apoptosis on HepG2 cells.

We used western blotting to detect the protein expression of HepG2 cells, treated with different dosages of lupeol. The expression levels of AKT and c-MET in HepG2 cells were very low. Therefore, the HepG2 cells were modeled first. The results (Figure 5) showed that compared with the WT group, the expression of AKT/mTOR-related proteins (p-AKT308 and p-AKT473) was significantly increased in HepG2 cells of the AKT/c-MET model group. After treatment, the expressions of AKT/mTOR-related proteins were significantly reduced in HepG2 cells. Compared to the free lupeol group, the expressions of AKT/mTOR-related proteins were also significantly reduced in HepG2 cells, treated with Gal-lupeol-L, indicating that Gal-lupeol-L was more effective than free lupeol.

### *In vivo* targeting studies

**Figure 6.**
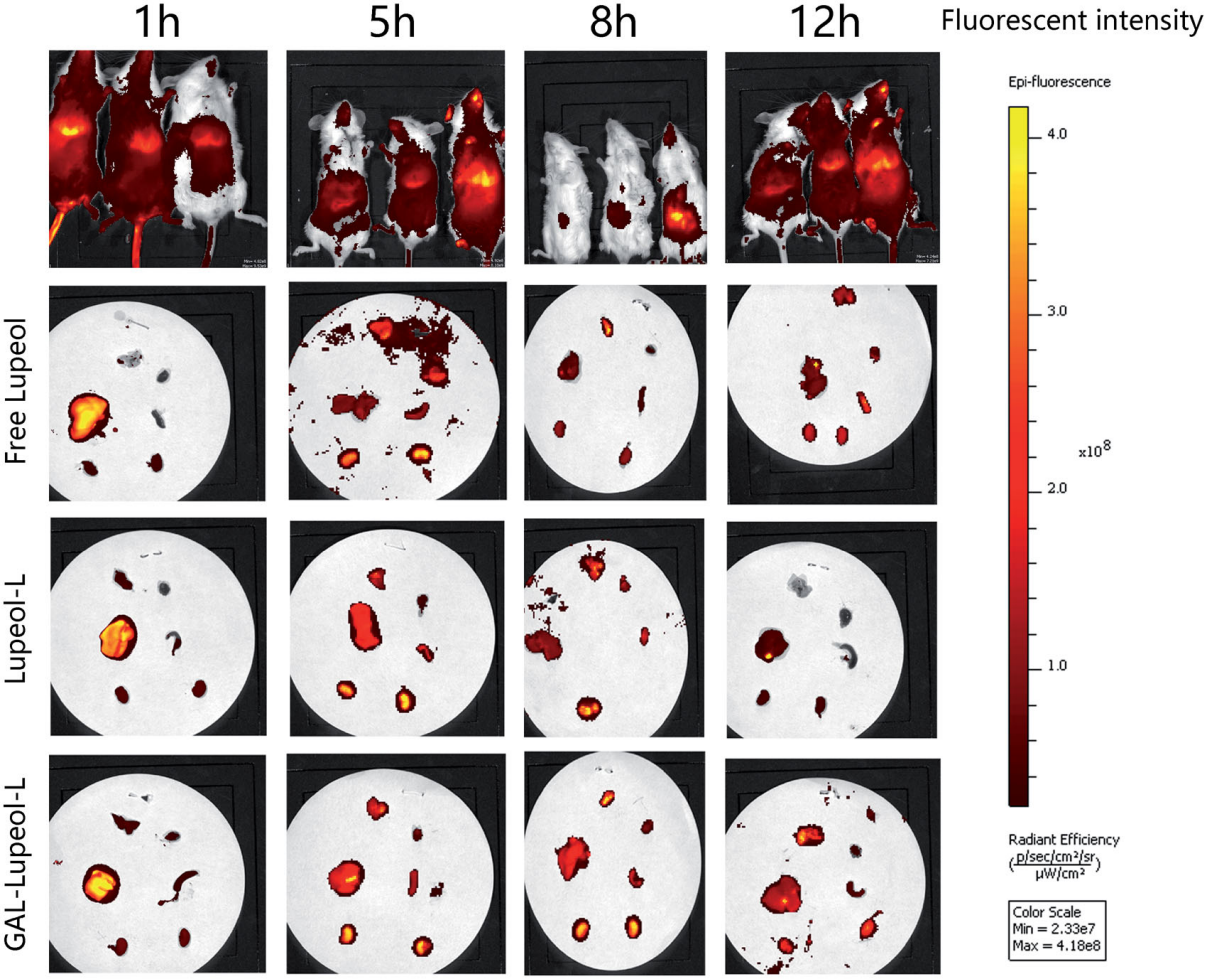
*In vivo* targeting studies result.

Figure 6 shows that the use of the NR infrared fluorescent probe can evaluate the distribution of the dosage-form. After injecting free NR, the time point of fluorescence distribution demonstrated that free NR had a very strong fluorescence distribution in the liver at 1 h. At 5 h, the *in vivo* imaging system suggests that the free drug has been metabolized in the liver, and the fluorescence distribution of the organs after dissection can also support the fluorescence distribution of the *in vivo* imaging. Among the non-targeted NR liposome-treated mice, the liver site showed a certain fluorescence in the first 5 h, which was metabolized from 8 h. After dissection, the non-targeted NR liposome group also demonstrated a strong fluorescence intensity in the first 5 h, and it was basically after 8 h. When it reached the kidney, the organs showed no obvious strong and weak characteristics, indicating that the non-targeted NR liposome was also quickly metabolized without any long targeting effect. In the GAL-NR-L group, the fluorescence intensity was seen in the liver part during live imaging from 1 to 12 h, and the fluorescence of the liver part accumulated from 1 to 12 h.

### *In vivo* pharmacodynamic experiments

**Figure 7.**
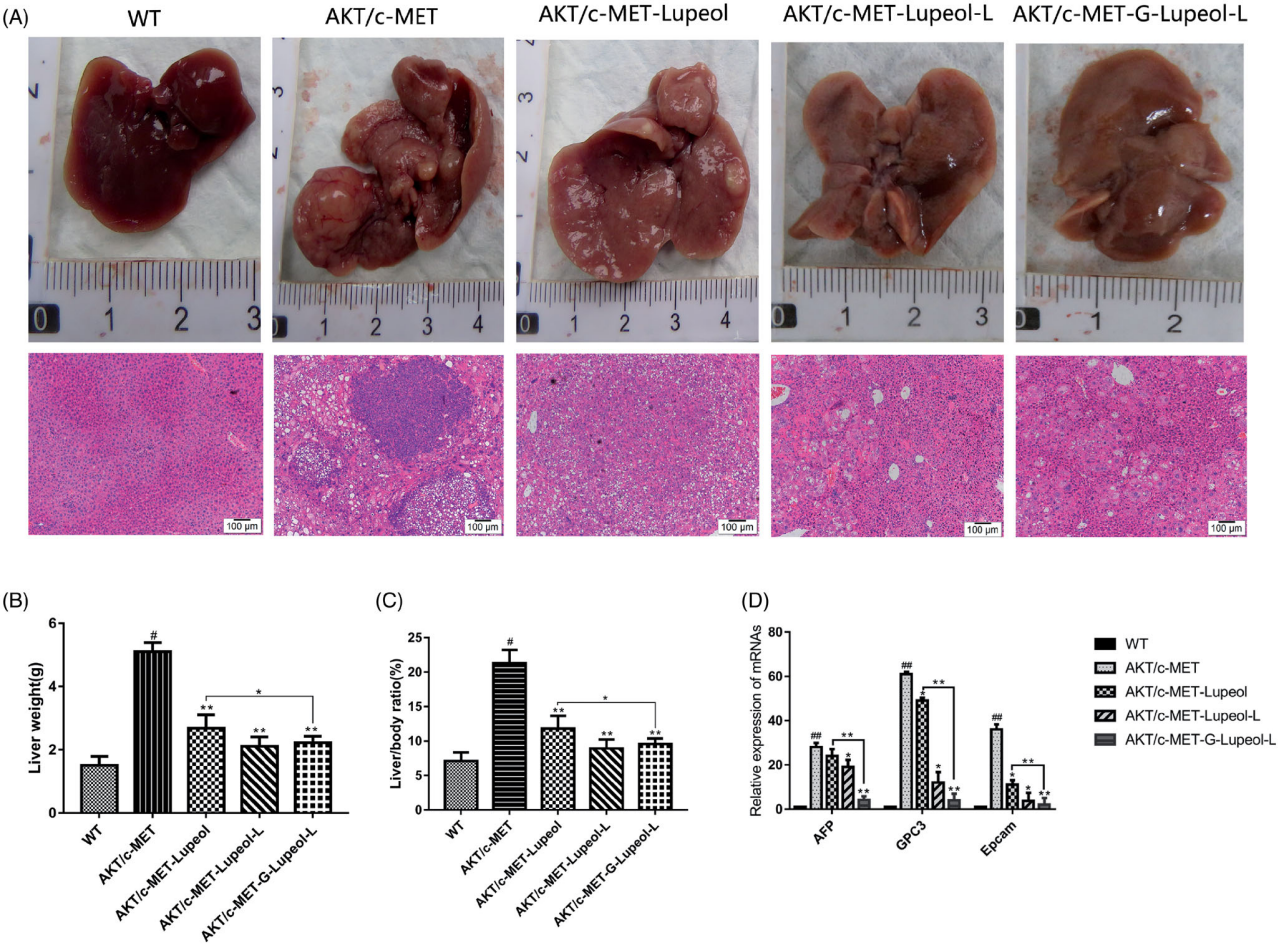
*In vivo* pharmacodynamic experiments. (A) Mouse liver morphology and HE result; (B) mouse liver weight; (C) mouse liver index; (D) qRT-PCR result.

After 3 weeks of Akt/c-Met plasmid transfection, FVB mice were administered with different dosage-forms of lupeol. The mice were killed four weeks later. The livers of each group were separated and weighed. Then, the livers were photographed and observed (Figure 7(A)). The representative images of the mice livers of different groups indicate that the liver of the WT group is bright red, its surface is smooth, no obvious tumor nodule is detected, and the whole liver is relatively uniform. The surface of the Akt/c-Met group is dark red, the liver surface is white and uneven, numerous similar granular substances are present with scattered nodules on the surface of the liver. Compared to the model control group (Akt/c-Met), the mice liver surface appeared light red, the sense of granule was reduced, the nodule condition was reduced obviously, and the liver size was significantly reduced, the appearance was more regular than that of the model group. Compared to the model group, the liver tumor node of mice in targeted group decreased significantly. The liver weight and liver index of different groups were statistically analyzed. Compared to the WT group, the liver index and liver weight of the model group (Akt/c-Met) were significantly increased (*P* < 0.05); compared to the model group (Akt/c-Met), different lupeol dosage-form groups were found that the liver index and liver weight (*P* < 0.05) of mice were decreased. Especially, the liver targeted group were significantly decreased than those in the free group (*P* < 0.05).

Histopathological analysis (Figure 7(A)) showed that the mice in the WT group had very clear liver lobules and nuclei. Meanwhile, the cytoplasm was relatively rich, and the hepatocytes around the central vein had a regular radial arrangement and clear cords. The liver lobules in the model group (Akt/c-Met) were completely destroyed, and numerous vacuoles were present in the hepatocytes. The nuclei, nucleoli, and nuclei were enlarged. We also observed more kernels, coarser chromatin, little cytoplasm, more mitotic phases, and obvious heteromorphism of hepatocytes in mice, treated with different dosage-forms of lupeol. Also, the liver lobules had better structure, and the vacuolation of hepatocytes had alleviated symptoms. Compared to the model group, the nucleus was significantly smaller, and the cytoplasm in the liver was fuller and smaller. The atypia of rat hepatocytes was obviously reduced. Compared to the control group, the structure of liver lobules, vacuoles and cytoplasm were clearer, more obvious, and more abundant in the liver targeting group than those in the free group.

QRT PCR was used to detect and analyze the liver cancer markers. Compared to the WT group, the mRNA expression levels of AFP, GPC3, and EpCAM in the model group (Akt/c-Met) increased significantly (*P* < 0.001). Compared to the model group, the mRNA expression levels of AFP, GPC3, and EpCAM in the mice liver of the free and nontargeted lupeol-treated group decreased significantly (*P* < 0.001). The mRNA expression levels of AFP, GPC3, and EpCAM were significantly lower in the mice liver of the targeted lupeol group (Figure 7(D)).

## Discussion

Drug-targeting can improve the efficacy of drugs, and reduce side effects because liver-targeted drugs can specifically deliver them to HCC areas (Watanabe et al., 2007; Bardania et al., 2017; Heath & Brown, 1989; Wu et al., 2015). In this research work, we designed and constructed a lupeol-loaded Gal-PEG-DSPE system, in which free lupeol and lupeol-L were used as control systems, and Gal-PEG-DSPE was used as the target ligand to deliver lupeol to the HCCs expressing ASGPR.

The successful combination of the liposomal surface of the lupeol and PEG chain has prolonged the circulation time by avoiding the clearance of the reticuloendothelial system and promoted the passive accumulation of tumor tissue through the EPR effect (Hagiwara et al., 2018; Maeda et al., 2013; Shen et al., 2015). PEG chain has also been used for conjugation. Therefore, PEG was selected as a drug carrier in this study. However, increasing the circulation time via PEGylation may not sufficiently enhance the therapeutic efficiency of the drug.

The strategy of modifying specific ligands through PEGylation can improve specific drug delivery through receptor-mediated binding. Due to their high affinity and rapid internalization in liver tumor cells, many researchers utilized PEG to specifically deliver drugs to the HCC cells via ASGPR (Zhou et al., 2012; Li et al., 2014; D'Souza & Devarajan, 2015; Oh et al., 2016). A recent study suggests that galactose may be a potential HCC ligand because it specifically binds to ASGPR (Weeke-Klimp et al., 2007; Wei et al., 2012; Mishra et al., 2013). Galactose can effectively target the HCC cells through ASGPR endocytosis (Weeke-Klimp et al., 2007; Wei et al., 2012; Mishra et al., 2013). Therefore, galactose is employed as an ASGPR targeting ligand. Through the combined effect of passive and active targeting mechanisms, the designed lupeol-loaded liposome system may improve the therapeutic efficacy of the HCC cells.

The Gal-lupeol-L system shows favorable physical and chemical properties, including uniform particle size, narrow particle size distribution, negative charge, high EE, and sustained drug release. The negative liposomes with particle size less than 200 nm demonstrated longer circulation time due to the weak interaction with serum proteins, and the accumulation of tumor cells had increased through the EPR effect (Zhao et al., 2011; Cai et al., 2012). Gal-lupeol-L system displayed spherical or elliptical vesicles with an average particle size of 131 nm and a zeta potential of −15 mv. The small particle size and negative charge of Gal-lupeol-L provided favorable conditions for HCC transport. We selected 15% sucrose as the lyophilization protective agent while preparing liposomes, and they could be preserved for six months at 4 °C. The appearance showed no significant change, and the encapsulation rate and particle size were within the qualified range.

To confirm the targeting ability of Gal-lupeol-L to HCC cells, we compared lupeol-L and free lupeol as controls. The cell uptake of nanoparticles may be affected by size, shape, material, cell type, and surface receptors (Luo et al., 2013). The same nanoparticles may enter different types of cells by different uptake mechanisms. Previous studies have also reported that galactose can be transported into HCC cells by ASGPR. Endocytosis may be induced by galactose (Warczynski et al., 1997; Peng et al., 2007; Dong et al., 2017). In this work, the analysis of living cell imager showed that in HepG2 cells, the uptake of Gal-lupeol-L was significantly higher than that of lupeol-L and free lupeol (*P* < 0.05), but not in the negative cells (*p* < 0.05). Of course, this study is incomplete, and the specific endocytosis of Gal-lupeol-L in different HCC cell-lines should also be explored. Similarly, the effect of nanoparticles on cytotoxic apoptosis largely depends upon the solubility and the release of substances, toxic drugs, cell types, and incubation time (Wei et al., 2012). In this work, to confirm the apoptotic effect of Gal-lupeol-L on HepG2 cells, free lupeol and lupeol-L was the control drugs. The results showed that the apoptotic effect of Gal-lupeol-L on HepG2 cells was significantly higher than that of other control groups (*P* < 0.05), which was confirmed by the results of the cell uptake study.

Akt, an important regulatory protein, protects cells from various apoptotic stimulations and regulates cell proliferation and cell cycle (Gedaly et al., 2012; Fang et al., 2017; Fu et al., 2019). It is the main regulatory molecule of the PI3K (phosphoinositide-3 kinase) pathway. Blockage of Akt signal transduction will lead to programmed cell death and inhibition of tumor cell growth (Wang et al., 2013; Sui et al., 2015; Jiang et al., 2017; Sun et al., 2019). We used Western blotting to measure the expression of Akt in HepG2 cells, treated with different dosage forms of lupeol, and found that the expression of Akt in HepG2 cells was very low. Therefore, HepG2 cells were modeled at first, and then the same dose of lupeol was administered in different dosage forms. The results showed that lupeol could inhibit p-Akt and p-Akt, and the inhibition of Gal-lupeol-L was the strongest.

High-pressure tail vein transfection technology can effectively establish the liver cancer model. It can solve some shortcomings of the above animal model of HCC, because of its low cost, short cycle, and simple operation. This technique can be used to study multiple signaling pathways. It is a mixture of sb (Sleeping Beauty transposon) and two plasmids, i.e., Akt and c-Met (1:10–1:25), diluted with normal saline (the volume of saline is equal to 10% of the weight of a mouse), and injected into the tail vein of mice rapidly (Chen & Calvisi, 2014). After high-pressure transfection, about 40% of hepatocytes will be transfected into plasmids. Once the oncogene is stably expressed in mouse hepatocytes, it will eventually lead to tumor formation. In this study, different dosage forms of lupeol were administered from the fourth week. The results revealed that the liver index and the weight of Gal-lupeol-L in Akt/c-met-induced HCC mice were significantly lower than those in the free group and nontargeted group (*P* < 0.05). Compared to the free lupeol group, the liver lobule structure of the Gal-lupeol-L group was clearer, the vacuole was reduced significantly, and the cytoplasm was more abundant. In the Gal-lupeol-L group, the mRNA expression of AFP, GPC3 and EpCAM decreased significantly (*P* < 0.01). The previous studies have also demonstrated that galactose can be transported into HCC cells by ASGPR. After 12 h of Gal-NR-L injection, the fluorescence in the liver area was still strong. After 1, 3, 5, and 12 h of Gal-NR-L injection, FVB mice were killed and then their hearts, liver, spleen, lungs, and kidneys were collected for imaging. The results showed that Gal-NR-L had a longer accumulation time in the liver than NR-L and free NR.

## Supplementary Material

Supplemental Material
